# Toxic Epidermal Necrolysis in Pregnancy: A Case Report

**DOI:** 10.7759/cureus.101492

**Published:** 2026-01-13

**Authors:** Anisha Choudhary, Murari Bharadwaj, Archana Barik, Vinita Singh

**Affiliations:** 1 Obstetrics and Gynecology, Tata Main Hospital, Jamshedpur, IND; 2 Anesthesia, Tata Main Hospital, Jamshedpur, IND

**Keywords:** cutaneous eruptions, drug reaction, pregnancy, stevens-johnson syndrome, toxic epidermal necrolysis

## Abstract

Stevens-Johnson syndrome (SJS) and toxic epidermal necrolysis (TEN) are rare, severe mucocutaneous adverse drug reactions associated with significant morbidity and mortality. SJS or TEN during pregnancy is rare and presents with unique diagnostic and management challenges, with potential risks to both the mother and fetus. We report a case of a 25-year-old primigravida at 29 weeks of gestation who presented with widespread cutaneous eruptions following ingestion of an over-the-counter herbal medication for headache. Clinical examination revealed extensive erythematous macules involving the face, trunk, limbs and genital region. Based on characteristic clinical features, extent of epidermal detachment, and temporal association with drug exposure, a diagnosis of TEN was established. The patient went into spontaneous preterm labor and delivered a male neonate vaginally. Multidisciplinary management with systemic corticosteroids and supportive care resulted in gradual clinical improvement and complete re-epithelialization. Both the mother and neonate had favorable outcomes. This case highlights the importance of early clinical recognition of TEN in pregnancy and emphasizes that even herbal and alternative medications may precipitate severe cutaneous adverse reactions. Prompt withdrawal of the offending agent, multidisciplinary supportive care, and individualized obstetric management are essential to optimize maternal and fetal outcomes.

## Introduction

Stevens-Johnson syndrome (SJS) and toxic epidermal necrolysis (TEN) represent uncommon but severe and potentially life-threatening mucocutaneous adverse reactions, most often precipitated by drug exposure. These conditions are characterized by purulent conjunctivitis, stomatitis with mucosal necrosis, widespread purpuric macules, and systemic manifestations. They are differentiated primarily by the extent of epidermal detachment: SJS involves less than 10% of the body surface area (BSA), TEN involves more than 30%, and cases with 10-30% detachment are categorized as SJS/TEN overlap [1,2].

The reported incidence of SJS and TEN is approximately two to seven cases per million population annually, although variations exist across different geographic locations and patient populations [3]. Increased risk has been observed among elderly populations, immunocompromised individuals such as HIV positive patients and patients with connective tissue disorders [4]. A female predominance has been reported in the literature, with a reported female-to-male ratio of approximately 2:1 [5]. Drug exposure remains the most common triggering agent, particularly medications such as allopurinol, antiepileptics, sulphonamide antibiotics, certain antiretrovirals like nevirapine, and nonsteroidal anti-inflammatory drugs [6]. In a subset of patients, however, no clear offending agent can be identified [7].

Pregnant women are at a higher risk of developing SJS and TEN possibly due to physiological immunological alterations and a relative immunocompromised state. Reported maternal and fetal complications include preterm delivery, low birth weight, neonatal respiratory distress, postpartum infections, and long-term genital tract sequelae such as stenosis or adhesions [6]. Despite these risks, published data describing SJS and TEN in pregnancy remain scarce. We report a rare case of TEN occurring in a pregnant woman at 29 weeks of gestation, suspected to be triggered by the intake of a herbal medication for headache.

This case report aims to highlight a rare and clinically significant occurrence of TEN in pregnancy, potentially associated with the use of herbal remedies. Given the altered immunological state of pregnancy and the widespread perception of herbal medications as safe, this report underscores an important yet underrecognized risk, thereby contributing to existing literature and emphasizing the need for cautious use of non-prescribed remedies during pregnancy.

## Case presentation

A 25-year-old primigravida at 29 weeks period of gestation (POG) presented to our hospital with complaints of lower abdominal pain for one day and widespread skin eruptions involving the face and body for the past four days. She had been receiving routine antenatal care at a primary healthcare centre and was diagnosed with gestational diabetes mellitus, for which she had been prescribed metformin 500 milligrams twice daily for the past six weeks. She gave a history of taking an over-the-counter herbal medication for headache six days back, following which she developed skin rashes that progressively worsened over the next three days.

**Figure 1:**
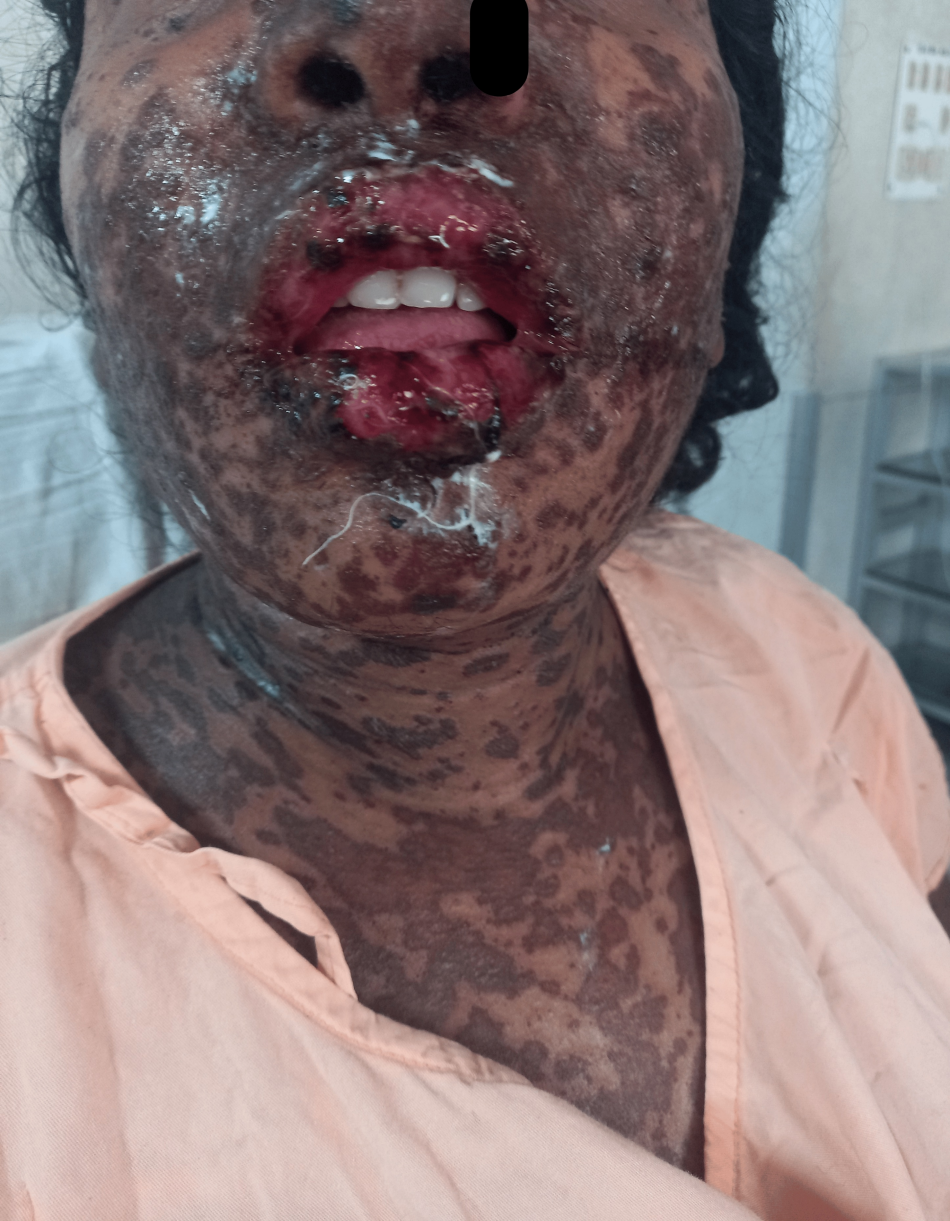
Erythematous lesions on the face and neck of the patient

On admission, she was found to be afebrile and clinically dehydrated. Her vital parameters included a pulse rate of 114 beats per minute, blood pressure of 94/60 mmHg, respiratory rate of 20 breaths per minute, and oxygen saturation of 98% on room air. Cutaneous examination revealed widespread erythematous macules distributed over the face and body including the genital region, with hemorrhagic crusting and erosions of the lips (Figure 1). Neurological and cardio-pulmonary examinations were within normal limits. Obstetric examination revealed a uterus corresponding to 30 weeks POG, with regular uterine contractions lasting for 20 seconds. The fetus was in cephalic presentation with a fetal heart rate of 132 beats/minute.

The patient was started on intravenous fluids and her initial baseline laboratory investigations were sent. Approximately one hour after admission, the patient had spontaneous rupture of the membrane. Pelvic examination revealed her cervix was five centimeters dilated with 70% effacement. She progressed rapidly and delivered a male baby weighing 1.35 kilograms. The APGAR (Appearance, Pulse, Grimace, Activity, and Respiration) score was 7/10 at one minute of birth [8]. The neonate was transferred to the neonatal intensive care unit (NICU) for further management. Initial laboratory results of the patient demonstrated anemia, thrombocytosis, elevated C-reactive protein (CRP), raised blood urea levels, and deranged hepatic enzymes, as summarized in Table 1.

**Table 1:** Laboratory investigations of the patient on the day of admission TLC: total leukocyte count; ALT: alanine transaminase; AST: aspartate aminotransferase; ALP: alkaline phosphatase; CRP: C-reactive protein; RBS: random blood sugar

Parameter	On admission	Normal range
Hemoglobin (gm/dl)	9	11.5-16.5
TLC (cells per mm^3^)	9,800	4,000-11,000
Platelet (cells per mm^3^)	454,000	150,000-410,000
Prothrombin time (seconds)	13.6	11-16
International normalized ratio	1	0.8-1.2
Total bilirubin (mg/dl)	1.32	0.2-1
Direct bilirubin (mg/dl)	0.14	0.1-0.5
ALT (U/L)	128	5-40
AST (U/L)	258	5-45
ALP (U/L)	202	35-125
Serum creatinine (mg/dl)	1.13	0.5-1.5
Blood urea (mg/dl)	50.9	15-40
Serum sodium (mEq/L)	132	136-146
Serum potassium (mEq/L)	3.8	3.5-5.5
Serum chloride (mEq/L)	96	95-108
CRP (mg/L)	21.06	0-5
RBS (mg/dl)	188	126-200

Based on the clinical presentation and progression, a diagnosis of TEN was established, with the suspected precipitating factor being the herbal medicine she took for her headache. Disease severity was assessed using the severity-of-illness score for toxic epidermal necrolysis (SCORTEN) scoring system, a validated prognostic tool for TEN [9]. Based on the patient’s clinical and laboratory parameters, the SCORTEN score was calculated to be 2, corresponding to an estimated mortality risk of approximately 12.1% (Table 2).

**Table 2:** Severity-of-illness score for toxic epidermal necrolysis (SCORTEN) The calculation of the score and its correlation with the fatality rate is as follows: the fatality rate is depicted in percentage. 0 to 1 = 3.2%, 2 = 12.1%, 3 = 35.3%, 4 = 58.3%, ≥5 = >90%. The score of the present case was 2 which comes to around 12% fatality rate.

Risk factor	Score	
	0	1
Life span in years	Less than 40 years	More than 40 years
Associated malignancy	Not present	Present
Pulse rate/minute	Less than 120	More than 120
Blood urea (up to 28 mg/dL is normal)	Normal	Elevated
Damage body (surface in percentage)	Less than ten	More than ten
Bicarbonate levels (20 mEq/L is normal)	Less than 20 mEq/L	More than 20 mEq/L
Serum glucose	≤ 250 mg/dL (≤ 13.88 mmol/L)	> 250 mg/dL (> 13.88 mmol/L)

Laboratory abnormalities, including elevated inflammatory markers and deranged metabolic parameters, contributed to the calculated SCORTEN score, which guided prognostic assessment and reinforced the need for intensive supportive care and multidisciplinary management. It also helped us to inform the patient and her relatives about the increased morbidity and mortality risks. A coordinated multi-disciplinary approach involving medicine, dermatology, and ophthalmology department was sought. She was started on oral antibiotics (capsule amoxicillin 500 milligrams thrice daily), systemic intravenous corticosteroids (injection dexamethasone 8 milligrams twice daily), oral antihistaminic (tablet cetirizine 10 mg twice daily), paracetamol infusion and vitamin supplements. For eye care, moxifloxacin and methylcellulose eye drops were given four times a day. For oral care, Candid mouthwash, betadine gargle and chlorhexidine mouthwash were prescribed. Within one week of initiating treatment, the appearance of new lesions diminished, and the patient’s symptoms began to gradually improve. Dexamethasone injections were gradually tapered over the second week, and no additional lesions developed. Improvement in laboratory parameters paralleled the patient's clinical recovery, supporting the observed response to supportive care and systemic therapy. Subsequently, injectable medications were discontinued and replaced with a tapering course of oral prednisolone.

**Figure 2:**
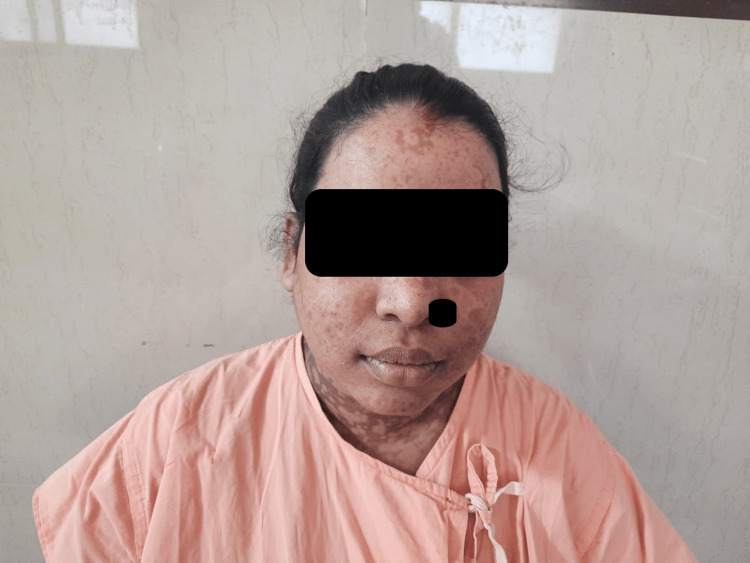
Healed lesions on the face and neck of the patient

**Figure 3:**
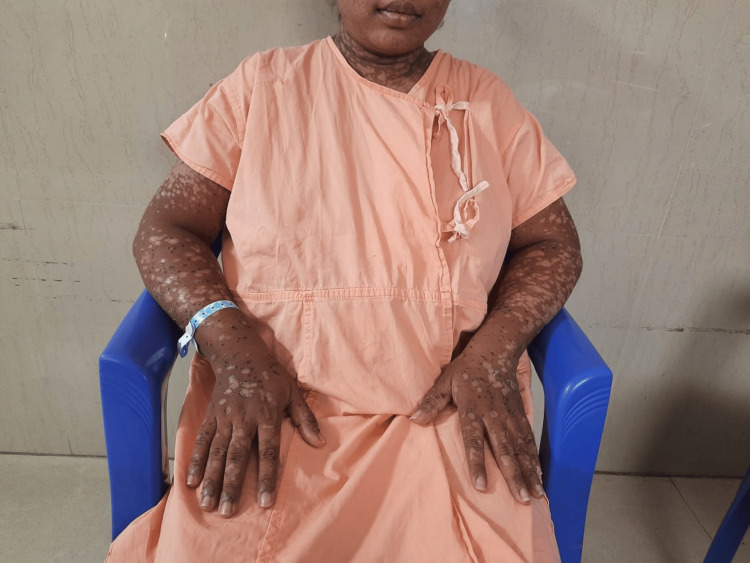
Healed lesions on the hands of the patient

Over the course of four weeks, the patient showed full re-epithelialization of affected areas. She was discharged in stable condition. On follow-up, the only residual finding was post-inflammatory hypo- and hyperpigmentation of the skin (Figures 2, 3). The neonate required short-term NICU care for observation, with no immediate complications noted during the hospital stay. The neonate was discharged along with the mother and remained asymptomatic on follow-up.

## Discussion

TEN is a rare but life-threatening disorder that belongs to the spectrum of severe cutaneous adverse reactions. It accounts for approximately 30%-50% of all serious drug-induced skin reactions [10]. SJS and TEN are now considered part of a single disease continuum, differentiated primarily by the extent of epidermal detachment, with SJS involving less than 10% of the BSA, TEN involving more than 30%, and intermediate cases classified as SJS/TEN overlap [1,2]. The most reported triggering agents are medications, followed by infections. Although uncommon, these conditions are associated with widespread epidermal necrosis and are considered a medical emergency due to their considerable morbidity and mortality, thus warranting prompt recognition and aggressive management [3]. The occurrence of SJS/TEN during pregnancy is exceptionally rare but poses significant risks to both the mother and the fetus. The underlying pathogenesis is believed to involve a cell-mediated immune response, in which cytotoxic T lymphocytes target keratinocytes expressing drug-related antigens, leading to widespread apoptosis and epidermal necrosis [3,4]. Pregnancy-related immunological modulation may further predispose susceptible individuals to such severe hypersensitivity reactions.

Medications remain the most implicated triggers of SJS/TEN. Drugs frequently associated with these reactions include antiepileptics, sulfonamides, non-steroidal anti-inflammatory drugs, and allopurinol [10]. Genetic susceptibility has also been demonstrated, particularly the association of the HLA-B*1502 allele with carbamazepine-induced SJS in Asian populations [11]. Additional risk factors include immunocompromised states (e.g., HIV infection), malignancies, connective tissue disorders, and certain infections such as herpes simplex virus or Mycoplasma pneumoniae [4]. In a large systematic review by Sharma et al., 177 cases of SJS/TEN during pregnancy were analyzed. The mean maternal age was 29.9 years, and the average gestational age at presentation was 24.9 weeks. Notably, a high prevalence of HIV infection was observed (85%), highlighting its role as a major predisposing factor. Antibiotics and other medications, including tocolytics and iron supplements, were also identified as causative agents, although less frequently [6]. Reports by Patel et al. have documented SJS/TEN following exposure to antibiotics such as penicillin and cefixime, emphasizing the need for cautious prescribing practices during pregnancy [12]. In contrast, the present case is unusual as the reaction followed ingestion of a herbal preparation taken for headache relief. Although alternative medicines are often perceived as safe, sporadic reports have described severe adverse cutaneous reactions following their use [13,14], underscoring the importance of a detailed drug history that includes non-allopathic remedies.

Clinically, TEN often begins with a nonspecific prodrome resembling a viral illness, followed by rapid progression to widespread erythematous macules, blistering, and epidermal detachment, giving the appearance of scalded skin [3]. Mucosal involvement is a hallmark of TEN and frequently affects the oral, ocular, and genital mucosae, potentially leading to both acute and long-term complications. The genital manifestations may include vulvar bullae, ulcerations, vaginal bleeding, and risk of secondary infection. Rare and severe long-term gastrointestinal complications such as labial synechiae and vaginal strictures have also been reported [15]. The latent period between exposure to the triggering agent and onset of symptoms can vary from four days to 28 days. In the present case, symptom onset occurred within five days of herbal drug ingestion, supporting a temporal relationship between exposure and disease development. Our patient presented with widespread erythematous macules over her face, body and genital region with epidermal detachment involving more than 30% of the BSA and associated with severe mucosal erosions suggestive of TEN. The temporal association between the initiation of herbal remedies and symptom onset raises suspicion of a possible trigger; however, definitive causality cannot be established in a single-case report.

The diagnosis of SJS/TEN is primarily clinical, based on characteristic mucocutaneous findings, extent of epidermal detachment, and a compatible drug exposure history. Histopathological confirmation, while helpful in atypical cases, is not mandatory when the clinical presentation is classic [3]. Skin biopsy is usually performed in the absence of clear clinical findings or to differentiate from other mucocutaneous disorders. Early recognition is crucial, as prompt withdrawal of the offending agent significantly influences prognosis. In our patient, skin biopsy was not pursued due to the classical clinical presentation, rapid disease progression, and the need for urgent management in a pregnant patient, where invasive procedures were deemed unlikely to alter immediate clinical decision-making. Differential diagnoses such as acute generalized exanthematous pustulosis and bullous drug reactions were considered; however, the extent of epidermal detachment, mucosal involvement, and disease course favored a diagnosis of SJS/TEN.

Management of TEN is challenging due to its multisystem involvement. In addition to extensive skin loss, patients are at risk of complications such as sepsis, renal impairment, electrolyte disturbances, hepatic dysfunction, and respiratory failure. Early withdrawal of the offending agent and intensive supportive care remain the cornerstones of treatment. Wound care, fluid and nutrition repletion, pain management, and prevention or treatment of superinfections are the main pillars of supportive care in such patients. Although no single immunomodulatory therapy has been universally accepted as standard of care, systemic corticosteroids, intravenous immunoglobulin, cyclosporine, and tumor necrosis factor inhibitors have been used with variable outcomes [1,2,16]. Data regarding the use of these therapies during pregnancy are limited. Most published cases have reported the use of systemic corticosteroids or intravenous immunoglobulin, while experience with newer biologic agents in pregnant patients is lacking [6,15,16]. In our patient, systemic corticosteroids were initiated following multidisciplinary consensus, balancing potential benefits in early disease control against maternal-fetal considerations. Use of systemic corticosteroids along with comprehensive supportive care resulted in a favorable maternal outcome. Obstetric management in patients with SJS/TEN should be individualized. Vaginal delivery is generally preferred unless contraindicated by obstetric indications, severe genital involvement, or fetal compromise [17]. In our patient, spontaneous labor occurred in the absence of significant genital disease or fetal distress, allowing for a successful vaginal delivery.

Maternal complications associated with SJS/TEN include sepsis, multiorgan failure, fluid and electrolyte imbalances, and psychological sequelae [18]. Reported mortality rates for SJS in pregnant women range from 14% to 19% and may reach up to 30% in TEN [17]. Fetal risks reported in the literature include preterm birth, growth restriction, fetal distress, and, in severe cases, stillbirth [17]. Although transplacental drug exposure may affect the fetus, direct fetal involvement with SJS or TEN is exceedingly rare, with only one isolated case reported in the literature [19]. In our case, the patient went into spontaneous labor and had a preterm vaginal delivery; however, there was no clinical manifestation of the disease in the newborn, who was discharged in stable condition.

Preventive strategies focus on the avoidance of high-risk medications in pregnant women and meticulous history taking of any drug intake, including alternative herbal medicines. Increased awareness among clinicians regarding drug-induced cutaneous reactions during pregnancy is essential for prompt management and reduction in associated morbidity and mortality.

This report has limitations inherent to a single-case design. The findings cannot be generalized to all pregnant patients with SJS or TEN. Histopathological confirmation was not performed; however, the diagnosis was established clinically based on classical mucocutaneous features, extent of epidermal detachment, and a clear temporal association with drug exposure, which is consistent with accepted diagnostic standards. As the patient did not come for long-term follow-up after discharge, therefore assessment of delayed cutaneous, ocular, or developmental sequelae was not done. Despite these limitations, this case contributes meaningful clinical insight into a rare and potentially life-threatening condition occurring during pregnancy.

## Conclusions

TEN during pregnancy is an uncommon but potentially catastrophic condition requiring early recognition and prompt multidisciplinary management. This case underscores the importance of maintaining a high index of suspicion for severe cutaneous adverse drug reactions, even with medications perceived as safe, including herbal and alternative therapies. Thorough drug history taking, timely clinical diagnosis, immediate withdrawal of the suspected agent, and intensive supportive care remain the cornerstones of management. Individualized obstetric decision-making can result in favorable maternal and neonatal outcomes. As a single-case report, these findings should be interpreted cautiously and are intended to highlight clinical considerations rather than establish definitive causal relationships or generalizable conclusions. However, increased awareness and reporting of such cases are essential to improve understanding of this disease in pregnancy and to enhance maternal-fetal outcomes.
